# Nitrate of Silver in Purpura Hæmorrhacica

**Published:** 1889-09

**Authors:** 


### NITRATE OF SILVER IN PURPURA HÆMORRHACICA.

Certain authors divide purpura into two forms,—the sthenic and asthenic,—a distinction which may be of service in furnishing therapeutic indications. The sthenic form is generally recognized by the pulse and the general state of the patient, and it may be imagined that in this form the walls of the capillaries are ruptured, without any alteration in their structure, on account of the exaggerated pressure in the circulatory system. In this form, reducing treatment is evidently indicated, and it is claimed that diminishing the blood-pressure will produce curative results. By far the greater number of cases of purpura hæmorrhagica, however, are of the asthenic or cachectic form, of which the termination is so often fatal. In this form there is more or less diminution in the red corpuscles and the solids of the blood, with augmentation of the white corpuscles, the great danger being in the incessant tendency to hemorrhage, which finally leads to fatal prostration. In such cases the state of the patient calls for an improvement in the health of the patient, and to the building up of his strength rather than to depletion. As a consequence, vegetable acids, especially citric acid, mineral acids, hæmostatic astringents, all occupy the first rank as remedies which are indicated; ergot also, in subcutaneous injections in the form of ergotine, may likewise prove efficacious. To this list of remedies Dr. V. Poulet (*Bulletin General de Therapeutique*, May 30, 1889) would add nitrate of silver, since he believes that the admirable results which this remedy produces in the treatment of purpura would almost warrant attributing to this remedy the possession of some specific properties. In support of this view Dr. Poulet reports two cases of purpura, which, he stated, were taken from his record of numerous others. In the most of these the hemorrhage resisted the most varied and energetic forms of treatment, such as hypodermic injections of ergotine, tamponing the nasal passages, etc., and finally yielded to the internal administration of the nitrate of silver. In the second case the remedies employed were less numerous, but equally fruitless ; and here also nitrate of silver was especially efficacious, for in a short time all tendency to the formation of purpuric effusions disappeared, and the tendency to hemorrhages was entirely overcome. Dr. Poulet believes that nitrate of silver produces this effect in these affections by its action on the vasomotor supply of the capillaries. He administers one-sixth of a grain of nitrate of silver twice daily in pill form, taking care to give at least half an hour after eating.—*Therapeutic Gazette.*

